# N-Methyltransferase *CaASHH3* Acts as a Positive Regulator of Immunity against Bacterial Pathogens in Pepper

**DOI:** 10.3390/ijms23126492

**Published:** 2022-06-10

**Authors:** Ansar Hussain, Liu Kaisheng, Ali Noman, Muhammad Furqan Ashraf, Mohammed Albaqami, Muhammad Ifnan Khan, Zhiqin Liu, Shuilin He

**Affiliations:** 1 Key Laboratory of Applied Genetics of Universities in Fujian Province, Fujian Agriculture and Forestry University, Fuzhou 350002, China; ahtraggar@yahoo.com (A.H.); lksfujian@126.com (L.K.); mifnan@yahoo.com (M.I.K.); alinoman@gcuf.edu.pk (A.N.); furqan2210uaf@scau.edu.cn (M.F.A.); 2 Department of Plant Breeding and Genetics, Ghazi University, Dera Ghazi Khan 32200, Pakistan; 3 Department of Botany, Government College University, Faisalabad 38000, Pakistan; 4 State Key Laboratory of Subtropical Silviculture, Zhejiang A&F University, 666 Wusu Street, Lin’An, Hangzhou 311300, China; 5 Department of Biology, Faculty of Applied Science, Umm Al-Qura University, Makkah 21955, Saudi Arabia; mmbaqami@uqu.edu.sa

**Keywords:** *CaASHH3*, immunity, *Ralstonia solanacearum*, *Pseudomonas syringae*

## Abstract

Proteins with conserved SET domain play a critical role in plant immunity. However, the means of organization and functions of these proteins are unclear, particularly in non-model plants such as pepper (*Capsicum annum* L.). Herein, we functionally characterized *CaASHH3*, a member of class II (the ASH1 homologs H3K36) proteins in pepper immunity against *Ralstonia solanacearum* and *Pseudomonas syringae* pv *tomato* DC3000 (*Pst* DC3000). The *CaASHH3* was localized in the nucleus, and its transcript levels were significantly enhanced by *R. solanacearum* inoculation (RSI) and exogenous application of salicylic acid (SA), methyl jasmonate (MeJA), ethephon (ETH), and abscisic acid (ABA). Knockdown of *CaASHH3* by virus-induced gene silencing (VIGS) compromised peppers’ resistance to RSI. Furthermore, silencing of *CaASHH3* impaired hypersensitive-response (HR)-like cell death response due to RSI and downregulated defense-associated marker genes, including *CaPR1*, *CaNPR1*, and *CaABR1.* The *CaASHH3* protein was revealed to affect the promoters of *CaNPR1*, *CaPR1*, and *CaHSP24.* Transiently over-expression of *CaASHH3* in pepper leaves elicited HR-like cell death and upregulated immunity-related marker genes. To further study the role of *CaASHH3* in plant defense in vivo, *CaASHH3* transgenic plants were generated in *Arabidopsis*. Overexpression of *CaASHH3* in transgenic *Arabidopsis thaliana* enhanced innate immunity against *Pst* DC3000. Furthermore, *CaASHH3* over-expressing transgenic *A. thaliana* plants exhibited upregulated transcriptional levels of immunity-associated marker genes, such as *AtNPR1*, *AtPR1*, and *AtPR2*. These results collectively confirm the role of *CaASHH3* as a positive regulator of plant cell death and pepper immunity against bacterial pathogens, which is regulated by signaling synergistically mediated by SA, JA, ET, and ABA.

## 1. Introduction

Plants have evolved various distinctive mechanisms to cope with rapidly varying environmental conditions [1,2]. It has been observed that tolerance is enhanced after repeated exposure to similar environmental stress. This phenomenon of augmented tolerance against repeated exposure to same environmental stress is known as acclimation [3,4]. For instance, *Arabidopsis* plants after exposure to high temperature (38 °C) showed higher tolerance to subsequent high temperature stress (45 °C) as compared untreated plants with high temperature treatment [5,6].

Moreover, plants are exposed to biotic stresses, e.g., microbial pathogen attack in addition to abiotic stresses. Defense responses are induced locally by recognition of microbe-associated molecular patterns (MAMPs), via membrane proteins of plants named as pattern recognition receptors [7,8,9]. The epitope peptide flg22 is a model bacterial MAMP that is derivative of flagellin [10]. This basic defense system is known as pattern-triggered immunity (PTI) [11]. The PTI is induced by the expression of defense-associated genes and deposition of callose [12]. Additionally, systemic resistance is generally associated with a sensitization of stimulus reaction called priming [13,14]. Priming allows cells to counter very low levels of stress in more speedy and vigorous manner as compared to non-primed cells [15]. Usually, primed plants show quicker and stronger activation of several defense responses that are induced after microbial pathogen infection [16].

Histones are assembled with DNA to build nucleosomes that are a fundamental component of chromatin. The histone tails can be methylated post-transnationally at manifold lysine sites and diverse levels of methylation (mono, di, and tri) [17,18]. Histone H3 lysine 36 (H3K36) methylation is an evolutionarily conserved epigenetic mark normally supposed to be involved with active chromatin [19]. Thus far, enzymes that consign methyl groups on Lys36 residues have been expressed in various yeasts and animals, with different preferences for various states of methylation. The H3K36 methylation has also been involved in different processes such as transcription commencement, suppression of transcription, dose compensation, alternative splicing, DNA replication, and DNA repair [19]. At least six lysine residues on histone H3 (K4, K9, K27, K36, and K79) and on histone H4 (K20) are targeted by histone lysine methyltransferases (HKMTs) [20,21]. Except for H3K79, SET-domain-possessing HKMTs have the capability to transmit one or several methyl groups to the ε-nitrogen of specific lysine residues on histones [22,23].

Distinct biological processes are involved in plant development that are differentially affected by environmental conditions [24]. According to annotations in the Pfam and ChromeDB databases (http://www.chromdb.org/, accessed on 11 February 2022) and prior phylogenetic analyses, plants may have developed diverse epigenetic mechanisms against various environmental stresses during growth and development [25,26]. Epigenetic mechanisms such as covalent histone modifications play a critical role in activation or repression of transcription by producing more “open” or “close” chromatin configurations, respectively [27]. Generally, openness of chromatin enhances the genome accessibility to transcription machinery such as RNA polymerase II (RNAPII), thus triggering the transcription process. Therefore, primed genes maybe in an open chromatin configuration. Innate immunity priming is currently associated with the modifications of chromatin in promoter regions of WRKY TFs [28] and also SA- and PTI-associated genes [29,30].

Proteins with a conserved SET domain are found in different organisms including viruses, bacteria, archaea, and eukayota. On the basis of the annotation from Pfam and Chrom, DB 47, 37, and 35 SET domain proteins are found in *Arabidopsis*, rice, and maize, respectively [31]. Plant SET proteins are classified into seven classes on the basis of their phylogentic relationship, domain organization, and sequence homology [25]. The role of SET domain proteins is still needed to elucidate in non-model plants including pepper.

Pepper (*Capsicum annuum* L.) is an important agricultural crop worldwide. Pepper is an important member of the Solanaceae family. It is generally sown on uplands in temperate weather conditions and mostly challenged by various soil-borne pathogens and diseases during its life cycle. Diseases, i.e., phytophthora blight and bacterial wilts [32,33,34] are major reasons for poor production of pepper and great threat to economic losses, especially under high temperature and high humidity (HTHH) [35,36]. *Ralstonia solanacearum* is a destructive soil-borne bacterium and causes wilt disease, which threatens the production of >200 economically vital plants species, including pepper and tobacco [37]. The research on genetics of plants’ disease resistance against *R. solanacearum* will be extremely beneficial for producing new superior crop varieties.

In this study, we reported a member of class II (the ASH1 homologs H3K36) plant SET domain protein family in pepper, named *CaASHH3.* We characterized expression and functions of *CaASHH3* and found that it was induced by infection of *R. solanacearum* and application of various exogenous phytohormones, including SA, MeJA, ETH, and ABA. Transient over-expression of *CaASHH3* in pepper plants caused HR-like cell death and silencing of *CaASHH3* in pepper plants, which compromised the resistance against *R. solanacearum* infection. We also reported that *CaASHH3* over-expression in transgenic *Arabidopsis thaliana* confers higher innate immunity against the bacterial pathogen *Pseudomonas syringae* pv *tomato* DC3000 (*Pst* DC3000). These results infer that *CaASHH3* is a positive regulator of plant cell death and pepper immunity against bacterial pathogen *R. solanacearum* and *Pst* DC3000.

## 2. Results

### 2.1. Cloning and Sequence Analysis of the Full-Length cDNA of CaASHH3

**Figure 1:**
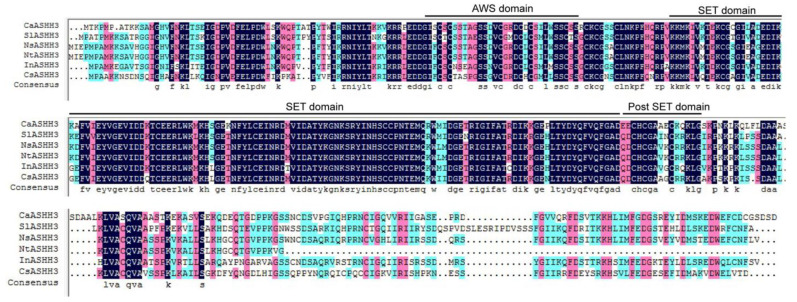
Multiple sequence alignment of sequences of proteins related to *CaASHH3*. Comparison of deduced amino acid sequence of *CaASHH3* with related proteins of *Solanum lycopersicum SlASHH3* (XP004228631.1), *Nicotiana sylvestris NsASHH3* (XP009773063.1), *Nicotiana tabacum NtASHH3* (XP016443214.1), *Ipomoea nil InASHH3* (XP019152077.1), and *Citrus sinensis CsASHH3* (XP006478510.1). Green shading indicates 50–75% similarity, red shading denotes 75–100% similarity, and black shading represents 100% similarity. Alignment was carried out by DNAMAN5.

The presence of SET-domain in *CaASHH3* protein implies its potential role in pepper immunity. The cDNA fragment of *CaASHH3* (*Ca methyl transferase*) that contained 1146 bp open-reading frame (ORF) was cloned (LOC107875209) by using gene-specific primers (Table S1). Its deduced amino acid sequence containing AWS-domain, SET-domain, and post-SET-domain was 381 amino acid residues in length and was classified into class II (the ASH1 homologs H3K36) [25] (Figure 1). The size of predicted protein was 42.64 kDa. The *CaASHH3* shares 83% of amino acid identities with SlASHH3, NsASHH3, and NtASHH3; 82% of amino acid identities with InASHH3; and 79% with CsASHH3 (Figure S1).

### 2.2. Transcriptional Expression Levels of CaASHH3 Were Upregulated by R. solanacearum Inoculation and Exogenously Supplied Phytohormones including SA, MeJA, ETH, and ABA

**Figure 2:**
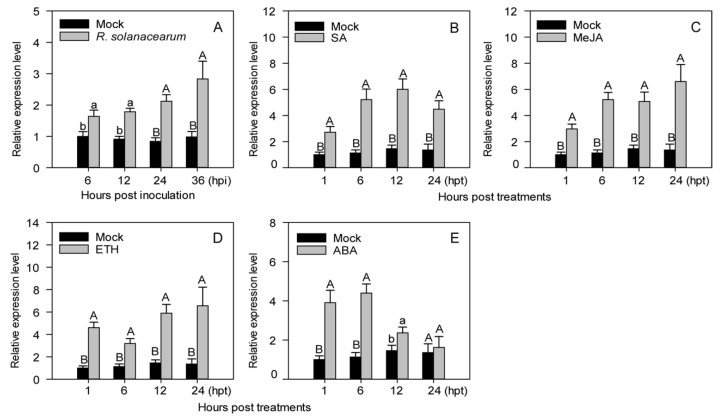
Real-time qRT-PCR analysis for relative transcript levels of *CaASHH3* in pepper leaves subjected to *Ralstonia solanacearum* and exogenous application of phytohormones. (**A**) qRT-PCR analysis of relative transcriptional expression levels of *CaASHH3* in pepper leaves at various time intervals after treatment with virulent *R. solanacearum* strain ‘FJC100301’. (**B**–**E**) qRT-PCR analysis of relative transcriptional expression levels of *CaASHH3* at various time intervals after treatment with 1 mM SA (**B**), 100 µM MeJA (**C**), 100 µM ETH (**D**), and 100 µM ABA (**E**). The relative transcriptional expression levels in *R. solanacearum*-treated and phytohormone-treated leaves were compared with mock plants (which normalized to a relative transcriptional expression level of 1). Error bars indicate the standard error. Different letters above the bars indicate significant differences between means from four biological replicates based on Fisher’s protected LSD test: uppercase letters represent *p* < 0.01; lower case letters represent *p* < 0.05.

The transcript levels of *CaASHH3* were significantly enhanced in leaves inoculated with *R. solanacearum* but not in the leaves without treatment (MgCl_2_) (Figure 2A). The increased *CaASHH3* transcript levels were maintained between 6 and 36 h post-inoculation (hpi) with the highest levels noticed at 36 hpi (Figure 2A). The results of transcriptional upregulation suggested that *CaASHH3* might play a critical role in pepper plants’ immunity against *R. solanacearum* infection.

We determined the role of these phytohormones in regulation of *CaASHH3* expression in pepper plants. Real-time qRT-PCR was used to assess the relative abundance of *CaASHH3* transcriptional expression of pepper plants supplied with exogenous SA, MeJA, ABA, and ETH. After application of 1 mM SA, *CaASHH3* transcript levels were significantly enhanced from 1 to 24 hpt (hours post treatment) as compared with the control (mock) plants, and the highest transcript level was recorded at 12 hpt (Figure 2B). The exogenous application of 100 µM MeJA significantly increased the transcriptional expression levels of *CaASHH3* from 1 to 24 hpt as compared to the mock plants and topped at 24 hpt (Figure 2C). The *CaASHH3* transcript levels were significantly increased after treatment with 100 µM ETH and maintained 1 to 24 hpt as compared to mock. The highest transcript level was observed at 24 hpt (Figure 2D). After application of 100 µM ABA, *CaASHH3* transcript levels were increased from 1to 24 hpt as compared to mock plants, and it was highest at 6 hpt (Figure 2E).

### 2.3. CaASHH3 Was Localized to the Nucleus

**Figure 3:**
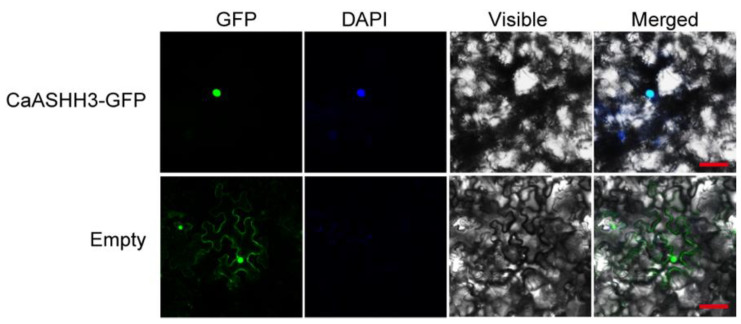
The localization of *CaASHH3* in the nucleus. Bars = 50 µm.

Sequence analysis using WoLFPSORT (http://www.genscript.com/psort/wolf_psort.html accessed on 17 February 2022) showed that the predicted *CaASHH3* protein possesses a putative nuclear localization signal. A *CaASHH3*–GFP fusion construct driven by the constitutive CMV 35S promoter was generated. This construct was transiently expressed in *N. benthamiana* leaves to confirm its localization. The construct *p35S*:GFP was used as a control. The results displayed the exclusive localization of *CaASHH3*-GFP to the nucleus. On the other hand, the *p35S*:GFP control was located in the multiple sites in subcellular portions, including the cytoplasm and nucleus (Figure 3). These results inferred that *CaASHH3* is localized to the nucleus.

The *CaASHH3* was exclusively found to be localized in nucleus of *N. benthamiana* leaves, transiently over-expressed in 35S:*CaASHH3*-GFP. The GFP signal for the control *N. benthamiana* leaves was found throughout the cell, including the nucleus, cytoplasm, and membrane. Green color indicates GFP, and blue color indicates DAPI staining of the nucleus. Cyan color indicates merger of GFP and the DAPI-stained nucleus. Images were taken by confocal microscopy at 48 hpi of *Agrobacteria* with 35S:*CaASHH3*-GFP construct and 35S-GFP construct (control).

### 2.4. CaASHH3-Silencing Compromised the Resistance of Pepper against R. solanacearum Infection

**Figure 4:**
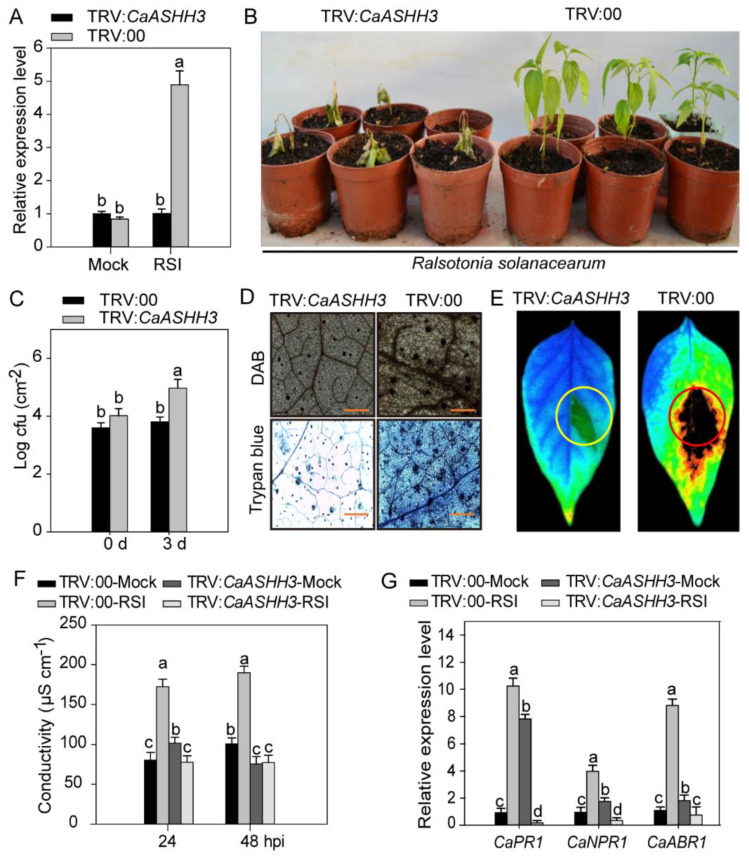
The *CaASHH3* silencing impaired the pepper plants’ resistance to *Ralstonia solanacearum* inoculation. (**A**) Real-time qRT-PCR analysis of *CaASHH3* transcript accumulation in *CaASHH3*-silenced (TRV:*CaASHH3*) and unsilenced plants (TRV:00) inoculated with *R. solanacearum*. (**B**) Phenotypic effect of *R. solanacearum* treatment on *CaASHH3*-silenced and unsilenced plants at 7 dpi. (**C**) Comparison of growth of *R. solanacearum* in *CaASHH3*-silenced and unsilenced plants inoculated with *R. solanacearum* at 0 and 3 dpi. (**D**) DAB and trypan blue staining in *R. solanacearum*-infected *CaASHH3*-silenced and unsilenced leaves at 2 dpi. Scale bar = 50 µm. (**E**) Assessment of cell death in *R. solanacearum*-infected, *CaASHH3*-silenced and unsilenced leaves under fluorescent modulation meter. (**F**) Measurement of electrolyte leakage as ion conductivity to check the cell death responses in leaf discs of *CaASHH3*-silenced and unsilenced plants at 24 and 48 hpi and without inoculation of *R. solanacearum*. (**G**) qRT-PCR expression of transcript levels of defense-associated marker genes in *CaASHH3*-silenced and unsilenced plants after inoculation and without inoculation of *R. solanacearum* at 24 hpi, respectively. The relative transcriptional expression levels of *CaASHH3* in mock-treated unsilenced plants were normalized to 1. Error bars indicate the standard error. Data represent the means ± SE from four biological replicates. Different letters indicate significant differences between means, as determined by Fisher’s protected LSD test: lowercase letters represent *p* < 0.05.

The loss-of-function experiments in pepper seedlings were performed to investigate the role of *CaASHH3* in immunity against pathogens by VIGS assay. We constructed TRV:*CaPDS*, which knocked down the *phytoene desaturase* gene (*PDS*), which induced a photo bleaching phenotype as an additional control to check the successful silencing of the desired gene. A total of 50 plants of TRV:00 and 50 plants of TRV:*CaASHH3* were acquired. Six plants were selected randomly for verifying the gene silencing efficiency by treating *R. solanacearum* strain ‘FJC100301’ by root inoculation. The results showed that transcript levels of *CaASHH3* were reduced to ≈30% in FJC100301-inoculated TRV:*CaASHH3* pepper plants as compared to TRV:00 plants, showing the successful silencing of *CaASHH3* (Figure 4A).

Ten plants of TRV:*CaASHH3* and TRV:00 were randomly selected and inoculated with *R. solanacearum* ‘FJC100301’ in the roots. At 7 dpi, strong wilting symptoms appeared on TRV:*CaASHH3*-silenced plants, but TRV:00-unsilenced plants exhibited only weak disease symptoms (Figure 4B). In the meanwhile, we observed significant magnification in the growth of *R. solanacearum* in *CaASHH3*-silenced plants, manifested by higher values of cfu at 3 dpi as compared to unsilenced plants (Figure 4C). Histochemical staining, i.e., trypan blue and DAB staining, were performed to evaluate the cell death and H_2_O_2_ accumulation of these *R. solanacearum*-infected, *CaASHH3*-silenced, and unsilenced plants. At 48 hpi, a clear deeper DAB staining (dark brown) and trypan blue color showing HR response and cell death in unsilenced plants leaves were detected. Nevertheless, DAB and trypan blue staining were distinctly reduced in *CaASHH3*-silenced leaves (Figure 4D).

The fluorescent modulation meter was used to take the photos of cell death in TRV:*CaASHH3* and TRV:00-unsilenced plants after infection with *R. solanacearum* strain ‘FJC100301’. We recorded obvious and strong cell death in the leaves of unsilenced plants and very weak or slight cell death in *CaASHH3*-silenced plants’ leaves (Figure 4E). We estimated electrical conductivity to analyze the cell necrosis severity caused by plasma membrane damage after RSI. The leaves of unsilenced plant exhibited stronger ion leakage as compared to *CaASHH3*-silenced plants at 24 and 48 hpi (Figure 4F).

The real-time qRT-PCR was used to detect the transcript abundance levels of defense-related genes involved in the response against pathogen inoculation. The transcriptional abundance of the defense-related pepper genes, i.e., *CaPR1*, *CaNPR1*, and *CaABR1* were reduced in *CaASHH3*-silenced plants’ leaves as compared to unsilenced plants at 24 hpi (Figure 4G).

### 2.5. Transient Over-Expression of CaASHH3 Caused HR-like Cell Death and Accumulation of H_2_O_2_ in the Leaves of Pepper Plants

**Figure 5:**
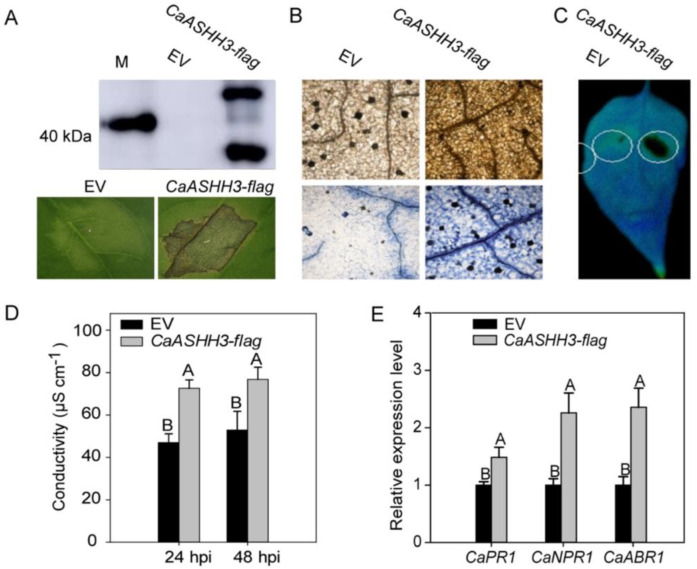
The HR-like cell death and transcriptional expression of immunity-associated marker genes induced by transient over-expression of 35S:*CaASHH3.* (**A**) The successful over-expression of *CaASHH3*-flag in pepper leaves as detected by Western blotting. (**B**) HR-like cell death triggered by transient over-expression of *CaASHH3*-flag detected by phenotype, DAB, and trypan blue staining at 2 dpi. Scale bar = 50 µm. (**C**) HR-like cell death caused by transient over-expression of *CaASHH3*-flag under fluorescent modulation meter. (**D**) Measurement of ion conductivity (electrolyte leakage) to evaluate the cell death response in leaf discs of peppers after over-expression of *CaASHH3*-flag at 24 and 48 h, respectively. (**E**) qRT-PCR analysis of the transcriptional expression levels of immunity-associated marker genes in *CaASHH3*-flag-expressed pepper leaves at 24 hpi. The relative transcriptional expression levels of empty-vector-treated control plants were normalized to 1. Error bars indicate the standard error of means. Data represent the means ± SE from four biological replicates. The different letters above the bars shows significant differences between means, as determined by Fisher’s protected LSD test: uppercase letters represent *p* < 0.01.

The successful expression of *CaASHH3* was confirmed by Western blotting experiment (Figure 5A). The DAB staining was performed to assess H_2_O_2_ production in pepper leaves transiently over-expressing *CaASHH3*. The DAB staining color was very weak or absent in the leaves transiently over-expressing the 35S:00-empty vector as compared to transiently over-expressing 35S:*CaASHH3* (Figure 5B). Trypan blue staining was used to identify the cell death. We observed no HR-mediated cell death in the leaves transiently expressing empty vector, while the leaves expressing 35S:*CaASHH3* construct induced a very strong and clear necrotic response (Figure 5B). The HR mechanism is used by plants for preventing the disease spread from infected to the healthy cells. This defense response includes the ROS production and cell death in infected tissues. We assessed involvement of *CaASHH3* in the regulation of HR mechanism. The *CaASHH3* was transiently over-expressed in leaves by infiltrating *A. tumefaciens* GV3101 harboring 35S:00 and 35S:*CaASHH3.* There was very strong HR-like cell death caused by over-expression of 35S:*CaASHH3*, while no or feeble HR-like cell death was caused by 35S:00-empty vector detected under modulation meter (Figure 5C).

Changes in electrical conductivity were recorded to assess the cell necrosis caused by plasma membrane damage in cells expressing 35S:*CaASHH3*. The pepper leaves transiently over-expressing 35S:*CaASHH3* displayed significantly high ion leakage at 24 and 48 hpi as compared to leaves expressing the 35S:00-empty vector (Figure 5D). We used the real-time qRT-PCR analysis to determine the transcript levels of known defense-related genes inclusive of ET biosynthesis-associated *CaPR1*, *CaNPR1*, and *CaABR1*. These results indicated steady increase in relative transcript levels of *CaPR1*, *CaNPR1*, and *CaABR1* during transient over-expression of *CaASHH3* as compared to 35S:00-empty vector at 24 hpi (Figure 5E). Keeping these results in view, it is confirmed that transient over-expression of *CaASHH3* can induce HR-like cell death and a play critical role in pepper plant immunity to pathogens.

### 2.6. ChIP-PCR Analysis to Test Chromatin Modification in Pepper

**Figure 6:**
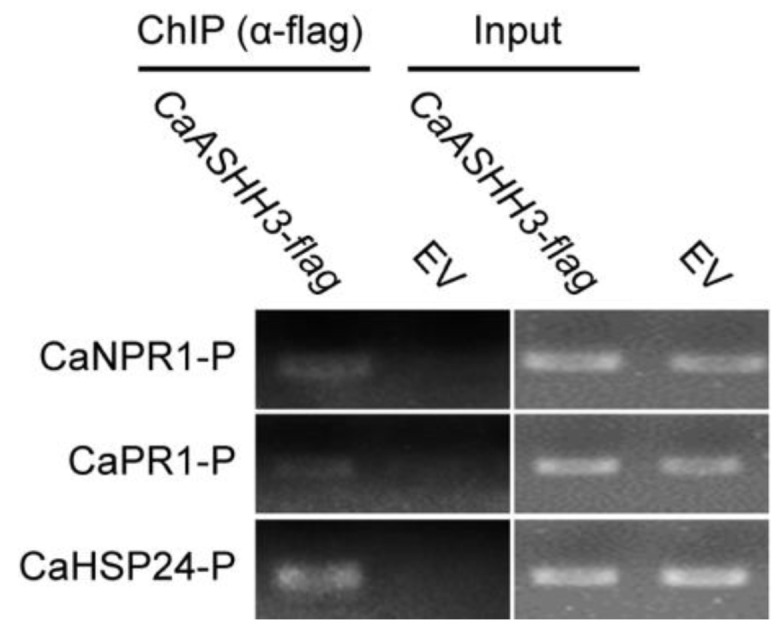
ChIP-PCR analysis indicating the interaction of *CaASHH3* and promoters of defense-related marker genes.

To evaluate possible histone modification in pepper plants after exposure to recurring stresses, chromatin immune precipitation (ChIP) was carried out by using histone-modification-specific antibodies. The chromatin was sheared into fragments of 300 to 500 bps in length, and then the protein–DNA complex was immunoprecipitated with HA antibody. The DNA was released and immunoprecipitated for further ChIP-PCR. The specific primers pair of known defense-associated genes including *CaNPR1*, *CaPR1*, and *CaHSP24* was used for ChIP-PCR, and immunoprecipitated fragments of DNA were used as PCR template. The results indicated that *CaASHH3* directly bind to histones (Figure 6)

The *CaASHH3* binds to histones of different defense-associated marker genes detected by ChIP-PCR. The *CaASHH3*-Flag was transiently over-expressed in pepper leaves and the chromatin was isolated; moreover, the DNA–protein complex was immuno-precipitated using anti-HA antibodies and adjusted to the same concentration, and PCR was performed using primer pairs based on *CaNPR1*, *CaNPR1*, and *CaHSP24*.

### 2.7. CaASHH3-OX (Over-Expression) in Transgenic *Arabidopsis* Plants Enhanced the Resistance to Bacterial Pathogen

**Figure 7:**
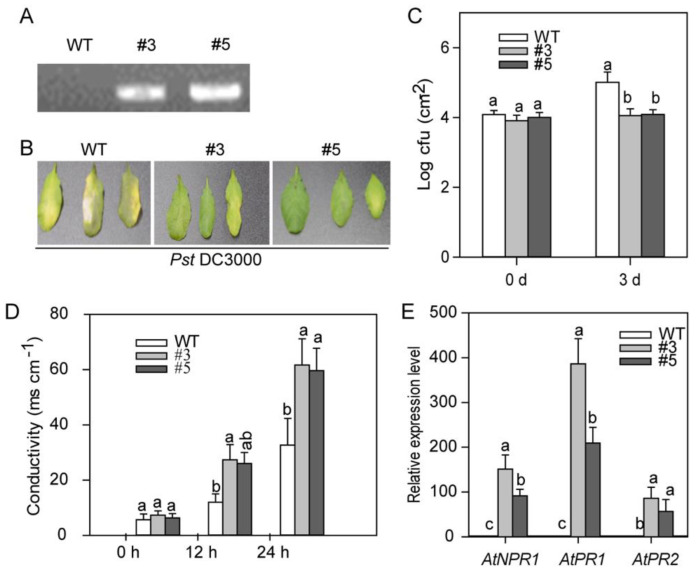
Enhanced resistance of *CaASSH3*-OX over-expressing transgenic *Arabidopsis* plants to *Pseudomonas Syringae* pv *tomato* (*Pst*) DC3000 infection. (**A**) Extraction of genomic DNA and PCR analysis of *CaASHH3* insertion in wild-type and *CaASHH3*-OX transgenic lines. (**B**) Disease symptoms of wild-type or *CaASHH3*-OX over-expressing transgenic plants inoculated with virulent *Pseudomonas syringae* pv *tomato* (*Pst*) DC3000 strains. Leaves of 4-week-old *CaASHH3*-OX transgenic and wild-type *Arabidopsis* plants were infiltrated by needless syringe with a suspension (10^5^ cfu mL^−1^) of virulent *Pst* DC3000. Disease symptoms were photographed at 3 dpi. (**C**) Bacterial growth in leaves of wild-type and *CaASHH3*-OX transgenic lines was investigated at 0 and 3 days after inoculation with virulent *P. syringae* pv *tomato* DC3000 (10^5^ cfu mL^−1^). (**D**) Quantification of electrolyte leakage from leaf tissues of *CaASSH3*-OX transgenic and wild-type plants inoculated with virulent bacterial pathogen *Pst* DC3000. Samples were harvested at 0, 12, and 24 h post-inoculation. (**E**) Real-time quantitative PCR of defense-related genes in leaves of wild-type and *CaASSH3*-OX transgenic plants after inoculation with virulent *P. syringae* pv *tomato* DC 3000 (10^5^ cfu mL^−1^) at 24 h (hours) time interval. The relative transcriptional expressions levesl of mock-treated plants were normalized to 1. Error bars indicate the standard error. Data represent the means ± SE from four biological replicates. Different alphabetical letters indicate significant differences between means, as determined by Fisher’s protected LSD test: lower case letters represent *p* < 0.05.

As the stable transformation is very hard to attain in pepper plants to further study the role of *CaASHH3* in plant defense in vivo. *CaASHH3* transgenic plants were generated in *Arabidopsis* that over-expressed the complete precursor under the control of constitutive 35S promoter (Figure 7). To confirm the *CaASHH3*-OX transgenic expression in independent homozygous T3 *Arabidopsis* lines, PCR analysis was performed by using genomic DNA extracted from *CaASHH3*-OX transgenic *Arabidopsis* plants and from wild-type plants (Col-0). The *CaASHH3* gene was amplified from two *CaASHH3*-OX plants, while PCR product was not detected for wild-type *Arabidopsis* plants (Figure 7A). The phenotype of *CaASHH3*-OX *Arabidopsis* plants was indistinguishable from wild-type plants under normal growth conditions. To check the disease resistance against bacterial pathogen, both wild-type and *CaASHH3*-OX *Arabidopsis* plants were inoculated with *P. syringe* pv *tomato* DC3000 by using needless syringe. Wild-type plants displayed very clear typical chlorotic symptoms on treated leaves at 3 dpi, whereas there were no obvious symptoms on *CaASHH3*-OX lines (Figure 7B). To verify that these disease symptoms reveal bacterial pathogen propagation in plants, the growth of bacterial pathogen was observed in plant leaves at 0 and 3 dpi. The results showed that there was increase in growth of pathogen in wild-type plants as compared with *CaASHH3*-OX plants, manifested by higher cfu values at 0- and 3-day intervals (Figure 7C). These results showed that *CaASHH3* over-expression confers enhanced resistance to plants against pathogen. Cell death related to HR response was measured by using electrolyte leakage assay. The *CaASHH3*-OX leaves showed high levels of electrolyte leakage as compared to wild-type plants at 0, 12, and 24 hpi with *P. syringe* pv *tomato Pst* DC3000 (Figure 7D).

To examine whether this enhanced immunity against the pathogen in *CaASHH3*-OX transgenic *Arabidopsis* plants is linked to the expression of SA-dependent *PR* genes, we evaluated the transcriptional expression of *AtNPR1*, *AtPR1*, and *AtPR2* in wild-type and *CaASHH3*-OX plants. The results indicated that the transcript levels of these defense-related marker genes were more abundant in *CaASHH3*-OX plants as compared to wild-type plants at 24 hpi with *P. syringe* pv *tomato Pst* DC3000 (Figure 7E). These results infer that SA-dependent immunity mechanism may play a critical role in *CaASHH3*-OX-enhanced disease resistance against bacterial pathogen attack.

## 3. Discussion

It is well known that plants are regularly exposed to pathogen attack throughout their entire life cycle. A great extent of the knowledge of how plants cope with pathogen attack has been yielded by the previous studies on model plants such as *Arabidopsis*, rice, or tobacco [2,24,38]. The SA or JA/ET signaling pathways are first to be turned on after plants encounter microorganisms [33,39,40]. SA-related signaling pathways are involved in resistance to bio-trophic pathogens, while JA/ET-dependent signaling pathways are involved in resistance against necro-trophic pathogens [41,42,43].

The DNA methylation and histone modifications play a crucial role in transcriptional regulations in plants [44,45,46]. Previous studies revealed that many chromatin and histone modifiers, as well as remodelers, have been involved in plant defense against biotic and abiotic stresses [46,47]. Immune responses are controlled by several epigenetic phenomena and histone modification post-transcriptionally, both synergistically and antagonistically over plant defense and transcription [48,49,50]. This is how different methylases, demethylases, and chromatin re-modelers play their role in various aspects of immunity. Some of them are positive and others are negative regulators of either particular aspects or of total immunity [49]. Many histone methyltransferases and de-methylases act as immunity regulators. Both types of enzymes have crucial role of regulation of histone methylation in plant defense. Some reported methyltransferases that are involved in plant immunity are ATX1, SDG8, and SDG25. Mutants of these methyltransferases are defective in basal defense, necro-trophic resistance, and SAR, respectively [26,51,52,53,54].

We cloned a *Capsicum annum* gene member of class II (ASH1 homologs H3K36) plant set domain protein family, named *CaASHH3*, that possesses methyl-transferase activity. The over-expression of *CaASHH3* induced the HR-like cell death response in pepper leaves, but loss of function experiments of *CaASHH3* compromised the plant resistance against pathogen and enhanced the cfu value in *CaASHH3*-silenced plants. Moreover, transgenic *Arabidopsis ASHH3*-OX showed more resistance as compared to wild-type plants. These data back the notion that *CaASHH3* acts as a positive regulator and plays a critical role in plant defense against pathogen. The involvement of *CaASSH3* in regulation of pepper immunity was further supported by the data that *CaASHH3* transcriptional expression levels were significantly upregulated by RSI.

Since genes upregulated upon exposure to certain stress are repeatedly found to have a role in response to that stress [55], we inferred that *CaASSH3* might act as a positive regulator in pepper’s resistance to RSI. The declined immunity of pepper plants was accompanied with amplified growth of inoculated *Ralstonia solanacearum* and downregulated [56,57], SA-associated *CaPR1* [57,58,59]; immunity-associated *CaNPR1* [60]; and *CaABR1* [61] under treatment to RSI. These studies support our hypothesis and are further supported by the results from loss-of-function experiments of *CaASHH3* by VIGS, gain-of-function experiments by transient over-expression, and *CaASHH3*-OX over-expression in transgenic *Arabidopsis*, respectively. The silencing of *CaASHH3* by VIGS significantly enhanced the vulnerability of pepper plants to RSI.

In comparison, transient over-expression of *CaASHH3* distinctively triggered HR-like cell death and accumulation of H_2_O_2_, consistent with upregulation of immunity-associated *CaPR1*, *CaNPR1*, and *CaABR1*. These findings of loss-of-function and gain-of-function experiments strongly recommend the role of *CaASHH3* as a positive regulator of cell death and immunity in pepper against bacterial pathogen. The similar plant HR has been observed in the case of CaZNF830 and CabZIP53 [32,39]. The distinguished plant responses in pepper and *Arabidopsis* confirmed the contribution of set of genes as well as transcription factors in plant innate immunity. The upregulation of defense marker genes is directly coupled with *CaASHH3* and other mentioned genes that ultimately support plant survival against *Ralstonia* and *P. syringae* pv DC 3000.

To further examine whether over-expression of *CaASHH3* bestows defense responses to heterologous plants against pathogen attack, we produced *CaASHH3*-OX transgenic lines in *A. thaliana* that were resistant to virulent *P. syringae* pv DC 3000 attack. Resistance responses in transgenic *Arabidopsis* plants included accumulation of ROS; damage of plasma membrane; necrotic disease symptoms; and upregulation of immunity-associated marker genes, including *AtNPR1*, *AtPR1*, and *AtPR2*. These results recommend that over-expression of *CaASHH3* gene in *Arabidopsis* increases basal defense or *R*-gene-mediated resistance against bacterial pathogen.

Phyto-hormones including SA, JA, and ET are involved in plant immunity signaling pathways. The SA triggers resistance against biotrophic pathogens, whereas JA and ET have a role in plant immunity against necrotrophic pathogens [2,62,63]. Production of hormones, i.e., SA, JA, and ET, is often coupled with ETI or PTI. These phytohormones can work synergistically or antagonistically depending upon their concentrations during defense singling [39,64]. These signaling components have a synergistic relationship during PTI. On the other hand, these signaling components have a compensatory relationship during ETI [40,65,66]. In our study, *CaASHH3* was constantly noticed as being induced by exogenous foliar application of phytohormones, including SA, MEJA, ETH, and ABA. The checked SA-, JA-, ET-, and ABA-based immunity-associated marker genes; SA-associated *CaPR1* and *CaNPR1* [67,68,69,70,71,72]; and ABA-associated *CaABR1* [73] were downregulated by silencing of *CaASHH3* by VIGS under RSI, whereas these HR-associated, SA-dependent, and ABA-dependent immunity-associated *PR* genes (*CaPR1*, *CaNPR1*, and *CaABR1*) were upregulated by transient over-expression of *CaASHH3* in pepper. Transcriptional expression levels of immunity-associated marker genes *AtNPR1*, *AtPR1*, and *AtPR2* were also significantly upregulated in *ASHH3*-OX-over-expressing transgenic *Arabidopsis* plants. These data indicate that *CaASHH3* takes part in defense signaling, which is mediated synergistically by SA, JA, and ABA, hence leading to PTI.

In chromatin immuno-precipitation assay, *CaASHH3* bound to promoter of immunity-associated marker genes *CaNPR1*, *CaPR1*, and *CaHSP24*, with these results suggesting that these immunity-associated marker genes are the target of *CaASHH3* and are directly regulated by *CaASHH3*.

## 4. Materials and Methods

### 4.1. Plant Materials and Growth Conditions

The seeds of pepper (*Capsicum annuum* L.) cultivar ‘GZ03’, *Arabidopsis thaliana* (ecotype ‘Col-0’), and *Nicotiana benthamiana* were obtained from pepper breeding group at Fujian Agriculture and Forestry University (www.fafu.edu.cn, accessed on 10 February 2022), Fuzhou, China. The seeds of pepper cv. ‘GZ03’, *A. thaliana*, and *N. benthamiana* were sown in a soil mixture comprising peat, moss, and perlite, [2/1(*v*/*v*)] in plastic pots (2.2 L), and placed in the growth chamber under controlled conditions of 25 °C, 60–70 µmol photons m^−2^ s^−1^, a relative humidity of 70%, and 16 h light/8 h dark photoperiod. The seeds of *Arabidopsis* were sterilized with 70% ethanol for 1 min prior to in vitro culture followed by treatment with 2% sodium hydroxide for 10 min. Ultimately, the seeds were washed with sterilized distilled water for 5 times and were sown in Petri dishes on solid Murashige and Skoog (MS) media for two weeks in growth chamber at a 16 h light and 8 h dark photoperiod.

### 4.2. Vectors Construction

The gateway technology was employed to construct the vectors. The full length ORF of *CaASHH3* (with or without terminal codon) was cloned into the entry vector pDONR207 by using BP reaction to construct satellite vectors. Then, LR reaction was employed to transfer the full length ORF of *CaASHH3* into destination vectors, including pMDC83, CD3688 (Flag-tag), pK7WG2, and CD3687 (HA-tag) to generate vectors for subcellular localization, over-expression, and ChIP assay, respectively. To construct vectors for virus-induced gene silencing (VIGS), a specific 310 bps fragment in 3′-untranslated region (UTR) of *CaASHH3* was selected, and the specificity was confirmed by BLAST against genome sequence in the database of CM334 (http://peppergenome.snu.ac.kr/) and Zunla-1 (http://peppersequence.genomics.cn/page/species/blast.jsp, accessed on 13 February 2022). The specific fragment was cloned into the entry vector pDONR207 by BP reaction and then transferred into the destination PYL279 vector by LR reaction. The primers used in vector construction are given in Table S1.

### 4.3. Pathogens and Inoculation Procedures

A virulent pathogen strain *R. solanacearum* ‘FJC100301’ was isolated from disease-infected pepper plants from Fujian province (China). Exudates of the stem and the stem vascular tissue portion from these plants were purified by using previously described tetrazolium chloride method [33,74]. Virulent *R. solanacearum* strain ‘FJC100301’ was cultured overnight in SPA medium (200 g potato, 20 g sucrose, 3 g beef extract, 5 g tryptone, and 1 L of ddH_2_O) at 200 rpm and 28 °C in thermo-control shaker. The cultivated *R. solanacearum* was then centrifuged at 6500 rpm and 28 °C for 10 min. The pellet was dissolved in distilled sterilized 10 mM MgCl_2_. Bacterial cell density was diluted to 10^8^ cfu mL^−1^ (OD_600_ = 0.8). To examine the effect of *R. solanacearum* inoculation (RSI) on transcript levels of *CaASHH3* and the resistance of pepper plants to RSI, pepper plants were infected with 10 µL *R. solanacearum* into the top third leaf with the help of a needleless syringe. The corresponding leaf samples were collected at the specified time intervals for RNA extraction and histochemical staining, including DAB and trypan blue staining. To examine the *CaASHH3*-silenced phenotype under *R. solanacearum* infection, pepper roots were wounded by giving minor cuts with a glass rod and then infected with *R. solanacearum*. After RSI, the plants were grown in a growth chamber under controlled conditions at 28 ± 2 °C, 60–70 µmol photons m^−2^ s^−1^, relative humidity of 70% and under a 16 h light/8 h dark photoperiod.

The virulent bacterial pathogen *P. syringae* pv. *Tomato* DC3000 (*Pst* DC3000) was also used in this study. To prepare bacterial pathogen suspension for the infection of *Arabidopsis* plants, bacterial strain was cultivated overnight at 200 rpm and 28 °C in LB media containing desired antibiotic. Afterwards, this suspension was centrifuged at 6500 rpm and 28 °C for 10 min. The liquid supernatant was discarded, and solid pellet was dissolved in distilled sterilized 10 mM MgCl_2_ and diluted to a 10^5^ cfu mL^−1^. Leaves of 5–6-week-old *Arabidopsis* plants were inoculated with *Pst* DC3000 pathogen suspension by using a needleless syringe. The infected plants were placed in a moist chamber for 18 h and then shifted to a growth room. The samples were harvested for examining phenotype, measuring electrolyte conductivity, colony forming units (cfu), and RNA extraction at the desired time points.

### 4.4. Treatment of Pepper Plants with Exogenously Applied Phytohormones

To study the response of phytohormones, healthy pepper plants at four-leaf stage were treated with foliar application of 1 mM SA, 100 µM MeJA, 100 µM ETH, and 100 µM ABA. Mock plants were treated with foliar application of sterilized ddH_2_O. The phyto-hormones and mock-treated samples were harvested for RNA extraction at specific time intervals.

### 4.5. Subcellular Localization Experiment of CaASHH3

The *Agrobacterium tumefaciens* strain GV3101 possessing 35S:*CaASHH3*-GFP constructs and 35S:GFP (used as control) were cultured overnight in LB media containing recommended antibiotics. The *Agrobacterium* cells were centrifuged, and the pellet was collected and dissolved in induction medium (10 mM MES, 10 mM MgCl_2_, 200 µM acetosyringone, (pH 5.6)) and diluted to OD_600_ = 0.8. *Agrobacterium* cells possessing the 35S:*CaASHH3*-GFP and 35S:GFP were injected into *N. benthamiana* leaves by using a needless syringe. Previously described 4,6-diamidino-2-phenylindole staining (DAPI) was carried out [75] to exhibit the nucleus. The GFP and DAPI fluorescence signals were perceived, and images were captured by utilizing a Leica fluorescence light microscope (Leica, Wetzlar, Germany) with an excitation wavelength of 488 nm, and an excitation wavelength of 405 nm with a 435–480 nm band-pass emission filter.

### 4.6. Virus-Induced Gene Silencing (VIGS) in Pepper Plants

For *CaASHH3* silencing experiments, the Tobacco Rattle Virus (TRV)-based VIGS system was used in pepper plants according to our previous studies [2,42,76]. *Agrobacterium tumefaciens* strain ‘GV3101’ containing pYL192 and pYL279-*CaASHH3* or pYL279 were cultured overnight in the shaker at 200 rpm and 28 °C. This *Agrobacterium* culture was centrifuged at 6500 rpm and 28 °C for 10 min. The pellet was collected by centrifugation and dissolved in induction medium (10 mM MES, 10 mM MgCl_2_, 200 µM acetosyringone, (pH 5.6)) and diluted to OD_600_ = 0.6. *Agrobacterium* strains with pYL192 vector and pYL279 vector, pYL279-*CaASHH3*, and pYL192-*PDS* (OD_600_ = 0.6) were mixed in a 1:1 ratio. This mixture was infiltrated into cotyledons of 2-week-old pepper plants using a needleless syringe. The *Agrobacterium*-injected plants were placed in a growth chamber at 16 °C in the dark for 56 h with 45% relative humidity and transferred into a growth room at 25 ± 2 °C, 60–70 µmol photons m^−2^ s^−1^ and a relative humidity of 70%, under a 16 h light/8 h dark cycle.

### 4.7. Agrobacterium-Mediated Transient CaASHH3 Over-Expression Assay

*Agrobacterium tumefaciens* strain ‘GV3101’ possessing 35S:*CaASHH3*-flag and 35S:00 vector (empty vector used as control) was cultured overnight to OD_600_ = 1 in LB media containing the corresponding antibiotics in a shaker at 200 rpm and 28 °C. Then, the cultured *Agrobacterium* was centrifuged, the supernatant was discarded, and the pellet was dissolved in induction medium (10 mM MES, 10 mM MgCl_2_, 200 µM acetosyringone, (pH 5.6)) and diluted to OD_600_ = 0.8. This suspension was injected into pepper leaves with the help of a syringe without a needle. The plants were kept in a growth room, and then samples were collected for further analysis at the desired time point.

### 4.8. Histochemical Staining

Trypan blue and 3,3′-diaminobenzidine (DAB) staining was performed for *CaASHH3* transiently over-expressed pepper leaves and VIGS-treated, *CaASHH3*-silenced and un-silenced pepper leaves inoculated with *R. solanacearum* according to a previously published method [8,57]. For trypan blue staining, treated pepper leaves were boiled in trypan blue staining solution (10 mL lactic acid, 10 mL glycerol, 10 mL phenol, 40 mL ethanol, 10 mL ddH_2_O, and 1 mL trypan blue) for 30 min, stored at room temperature for 8 h, followed by transfer into choral hydrate solution (2.5 g of chloral hydrate dissolved in 1 mL of distilled water), and these leaves were again boiled thrice to destain for 20 min each time. Finally, samples were kept in 70% glycerol. For DAB staining, the treated pepper leaves were dipped in 1 mg/mL of DAB solution and kept at room temperature overnight. Lactic acid/glycerol/absolute ethanol (1:1:3 (*v*/*v*/*v*)) solution was used to destain the DAB-stained pepper leaves and was placed in 95% absolute ethanol [77]. The images of DAB and trypan-blue-stained leaves were captured by camera and a light microscope (Leica, Wetzlar, Germany).

### 4.9. RNA Extraction and Real-Time qRT-PCR 

Total RNA was extracted from pepper leaves by using TRIzol reagent (Invitrogen, Carlsbad, CA, USA) and reverse-transcribed by using a Prime Script RT-PCR kit (TaKaRa, Dalian, China). To check the relative transcript levels of selected marker genes, qRT-PCR was carried out with specific primers (Table S2) according to the manufacturer’s instructions for the Bio-Rad Real-Time PCR system (Bio-Rad, Foster City, CA, USA) and SYBR premix Ex Taq II system (TaKaRa Perfect Real Time). Real-time qRT-PCR and corresponding data processing were performed as described previously [24,78].

### 4.10. Measurement of Ion Conductivity

Electrolyte ion conductivity (ion leakage) was measured according to a previously described method with slight amendments [9,79]. The six leaf discs (4 mm in diameter) were cut with a hole-puncher, then washed with sterilized ddH_2_O thrice and directly incubated in 10 mL of double distilled water. These discs were placed in a shaker with gently shaking (60 rpm) for 1 h at room temperature. The ion conductivity was assessed with a conductivity meter (Mettler Toledo 326 Mettler, Zurich, Switzerland).

### 4.11. Immunoblotting

Total protein from pepper samples was extracted by using protein extraction buffer as described earlier [39]. The extracted protein was incubated overnight with anti-HA agarose at 4 °C (Thermo Fisher Scientific, Waltham, Massachusetts, USA). Magnetic crack was used to collect the beads, and they were washed thrice by tris-buffered saline (TBS) and tween 20 (0.05%). Eluted proteins were observed using immunoblotting and by anti-HA-peroxidase (Abcam, Cambridge, UK).

### 4.12. ChIP Assays

The ChIP assays were accomplished as described in earlier studies [80,81]. Briefly, *Agrobacterium* cells possessing the construct 35S:*CaASHH3*-flag or 35S:00-flag were injected into the leaves at the eight-leaf stage. The treated leaf samples were collected at the required time interval, and extraction of chromatin and precipitation was conducted from these 5- to 6-week-old leaves (1.5 to 2 g of leaf tissue) treated with 1% formaldehyde. The chromatin was sheared to an average length of 300 to 500 bp by sonication and immuno-precipitated with antibodies against hemagglutinin (HA; Santa Cruz Biotechnology). A total of 10 mg of antibodies were used for ChIP analysis, washed with TBST buffer, and reverse cross-linked, and finally the DNA was eluted with sterilized ddH_2_O. The immuno-precipitated DNA was analyzed for enrichment of *CaASHH3* at the promoter region of target genes by using common ChIP-PCR. Every sample was quantified thrice. The primers used for ChIP-PCR analysis in ChIP assays are listed in Table S3.

### 4.13. Generation of CaASHH3-OX-Overexpressing Transgenic Arabidopsis Plants

The full-length *CaASHH3* cDNA sequence was cloned and integrated into the entry vector pDONR207 by BP reaction. Then, LR reaction was carried out to ligate this fragment into destination pK7WG2 vector. The vector pK7WG2 harboring the cauliflower mosaic virus 35S promoter–*CaASHH3* construct was transformed into *Agrobacterium tumefaciens* strain GV3101. *Agrobacterium*-mediated transformation was conducted to generate *CaASHH3*-OX-overexpressing transgenic *Arabidopsis* plants by using the floral dip method [82]. Seeds were harvested from these transgenic *Arabidopsis* plants and sown in Petri plates on MS agar media containing 50 µg mL^−1^ kanamycin to obtain independent transgenic lines. The PCR was carried out on confirming the insertion of *CaASHH3* cDNA in the genome of transgenic *Arabidopsis* plants.

## Data Availability

All the data are within the manuscript and its supporting information files.

## Supplementary Materials

The following supporting information can be downloaded at https://www.mdpi.com/article/10.3390/ijms23126492/s1.
