# Supplementary material for: N-Methyltransferase CaASHH3 Acts as a Positive Regulator of Immunity against Bacterial Pathogens in Pepper
Source: Int J Mol Sci. 2022 Jun 10;23(12):6492. doi: 10.3390/ijms23126492 (PMC9224371; doi:10.3390/ijms23126492)
Supplement: Supplementary file 1 [file ijms-23-06492-s001.zip › ijms-1729593-supplementary.pdf]

N-methyltransferase CaASHH3 Acts as aPositive Regulator of Immunity Against Bacterial Pathogens in Pepper

Ansar Hussain[1,2,+], Ali Noman[3], Muhammad Furqan Ashraf[4], Muhammad Ifnan Khan[1,2], Zhiqin Liu[1,*],Shuilin He[1,*]

[1]Key Laboratory of Applied Genetics of Universities in Fujian Province, Fujian Agriculture and Forestry University, Fuzhou, 350002, China

[2]Department of Plant Breeding and Genetics, Ghazi University, Dera Ghazi Khan, Pakistan

[3]Department of Botany, Government College University, Faisalabad, Pakistan

[4]State Key Laboratory of Subtropical Silviculture, Zhejiang A&F University, 666 Wusu Street, Lin'An, Hangzhou, 311300, China

* Correspondence: lzqfujian@126.com (Zhiqin Liu), shlhe201304@aliyun.com (Shuilin He);

SUPPLEMENTARY MATERIALS

Supplementary Figures

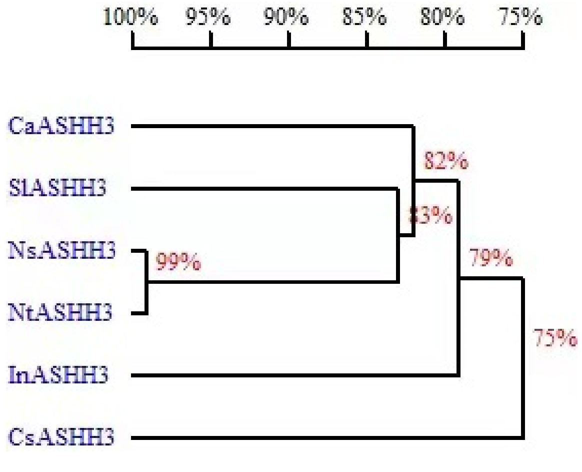

Supplementary Figure S1. Dendrogram presenting amino acid similarity of CaASHH3 and SET domains from other species

CaASHH3 shares 83% of amino acid identities with SlASHH3, NsASHH3 and NtASHH3, 82% of amino acid identities with InASHH3, and 79% with CsASHH3, respectively.

Supplementary Table S1. Pepper primers used for vectors' construction

Gene	Forward primer (5'-3')	Reverse primer (3'-5')	Size (bP)
CaASHH3[1]	ATGAAGCACAGTGGGGAAAA	TCATAGCTTCTGCTTGCATT	924
Ca ASHH3[2]	CGCCACCTCTACCAAAGAG	ACAATGTTGTGTCAGTCATCCA	310
Ca ASHH3[3]	ATGAAGCACAGTGGGGAAAA	TAGCTTCTGCTTGCATTCCA	924

1Primers for full length cloning of CaASHH3

2Primers for construction of TRV:CaASHH3vector

3Primers for construction of 35S:CaASHH3-GFP

Supplementary Table S2. Primers used in Real timeqRT PCR analyses

Gene	Accession number	Forward primers (5'-3')	Reverse primers(3'-5')	Size (bp)
CaASHH3[1]	LOC107875209	GACGGTGAGACAAGAATTGAC	ATAGCTTCTGCTTGCATTCC	219
CaPR1	AF348141.1	GCCGTGAAGATGTGGGTCAATGA	TGAGTTACGCCAGACTACCTGAGTA	108
CaNPR1	X61679.1	ACTTCTTCGCCGACGCCAAG	GCCAACACATTCACCAGAGCATC	190
CaABR1	CA524559	ATGACAGGCACAACAGAAGAAAAT	AATAAGTTATGACAGAGCCATTTT	148
CaActin	GQ339766	AGGGATGGGTCAAAAGGATGC	GAGACAACACCGCCTGAATAGC	225
18srRNA	EF564281	CCGGTCCGCCTATGGTGTGCACCGGTCGT	GCAGTTGTTCGTCTTTCATAAATCCAAG	285

C AA

1Specific primers to detect relative expression of CaWRKY30 designed according to the sequence in 3’UTR

Specific primers for marker genes

Supplementary Table S3. Primers used in ChIP experiment

Gene	GenBank Accession #	F_primer	R_primer
PNPR1-Tss	X61679.1	CAAAAAAATGGCAAACTCTC	AGGGAAAAGAAGAGGAGATA
PHSP24-Tss	HM132040	TGAGTTTCTCGAATCCTTTTTCCC	TTCGCCGACTTAGCTTCACG
PPR1-Tss	XM_016683907.1	AGTAGTGGCGGCTGTTATCG	AGCAGACTCTACAGGATTAT
PNPR1-CK	X61679.1	GAGCTAATAAATGAGCAGGT	CAATTATCCAGGAAAATGCT
PHSP24-CK	HM132040	TTTGACTCAATTTACTAGCG	CGTAAACTATTGATATTTTA
PPR1-CK	XM_016683907.1	ATATAAATCCTTATTATAAG	ACCAAGTGGTCCATAACGAT
